# Stability of hematological parameters of canine blood samples stored with citrate phosphate dextrose adenine-1 anticoagulated plastic vacutainers

**DOI:** 10.14202/vetworld.2019.449-453

**Published:** 2019-03-25

**Authors:** Okereke Henry Nnamdi, Udegbunam Rita Ijeoma, Nnaji Theophilus Okaforx

**Affiliations:** Department of Veterinary Surgery, Faculty of Veterinary Medicine, University of Nigeria, Nsukka, Nigeria

**Keywords:** anemia, blood, citrate phosphate dextrose adenine-1, plastic vacutainer, transfusion

## Abstract

**Aim:**

The effect of citrate phosphate dextrose adenine-1 on the hematological parameters of stored Nigerian indigenous dog’s blood with plastic vacutainer was investigated. This was done in view of determining the viability and stability of the studied parameters for blood banking purpose. Till date, there is no literature on the stability of whole blood of Nigerian indigenous dogs for blood banking purposes.

**Materials and Methods:**

A total of six apparently adult healthy dogs were sampled, and their blood was stored at 4°C and analyzed for their packed cell volume (PCV), hemoglobin (Hgb) concentration, red blood cell (RBC) count, total and differential white blood cell (WBC) count, platelet count (PC), mean corpuscular values (mean corpuscular Hgb [MCH], MCH concentration, and mean corpuscular volume [MCV]), blood potency of hydrogen (pH), and erythrocyte sedimentation rate (ESR) over a period of 14 days.

**Results:**

Significant changes were observed in some of the studied parameters. Of the 14 days’ study period, PCV, Hgb concentration, total WBC count, PC, and neutrophil count showed no significant (p≥0.05) difference until day 2 post-storage (PS), while RBC count, ESR, MCV, and lymphocyte count showed no significant (p≥0.05) variation up until day 3 PS. Blood pH showed no significant (p≥0.05) variation within 24-h PS but was significantly (p≤0.05) higher than obtained values on days 1, 3, 7, 10, and 14 PS.

**Conclusion:**

Based on our finding, we could suggest that hematological laboratories and hemotherapists could use canine blood stored in a plastic vacutainer for 2-3 days.

## Introduction

Whole blood degrades progressively during the weeks of refrigerated storage [1]. Hematological, biochemical, and physical properties of whole blood are adversely affected during storage by preservatives used, storage temperature, containers used, and frequency of gentle mixing during storage [2]. While practices vary from country to country, preservative solutions permitting shelf life as long as 7 weeks have been licensed [3]. These preservative solutions such as citrate phosphate dextrose adenine-1 (CPDA-1) are often contained in blood bags meant for the collection of blood for transfusion. It has been documented that blood stored in CPDA-1 anticoagulated bags remained stable for up to 35 days [1,3]. Sample stability is defined as the capability of blood to retain the initial value of a measured quantity for a defined period within specific limits when stored under defined conditions [3]. CPDA-1, a commonly used anticoagulant in blood bags, contains dextrose which supports continuing adenosine triphosphate (ATP) generation by glycolytic pathway while the citrate prevents coagulation by binding ionized calcium which is required for blood coagulation [2,4].

Blood transfusion is becoming a common practice for veterinarians in Nigeria. For this purpose, hemotherapy clinicians make use of CPDA-1 anticoagulated blood bags meant for human blood storage for blood collection and storage. These commercially available blood bags used for canine blood storage are for collecting up to 450 ml and 250 ml of blood for adults and infants, respectively. However, Nigerian indigenous dogs which are often used as donor dogs in our locality are of the small breed mostly weighing <10 kg. Therefore, since it is indicated that not >10% of an animal’s total blood volume is to be collected for transfusion [5,6], the volume of blood usually collected from these dogs does not fill the human blood bags. Thus, in our practice during blood transfusion, the amount of anticoagulant suitable for the volume of blood to be collected is retained in the blood bag while the excess is discarded.

In view of the above challenge associated with the use of human blood bags, there is a need to access an alternative storage medium such as sterile plastic vacutainer for blood banking purposes. This plastic medium will allow storage of small volumes of blood meant for transfusion. This study was conducted to evaluate the stability of canine blood stored in CPDA-1 anticoagulated plastic vacutainer.

## Materials and Methods

### Ethical approval

Animal studies were performed in conformity with the National Institutes of Health revised guidelines for laboratory animals’ care and use [7] and the University of Nigeria ethical codes and regulations for animal use.

### Animals

Six Nigerian indigenous adult dogs with an average mean weight of 11.5 kg aged between 2 and 4 years were used for this study. The dogs were procured from market in Nsukka Local Government Area. They were housed singly per each cage in the Department of Veterinary Surgery dog kennel and acclimatized for 30 days before the commencement of the experiment. They were fed with commercial dog food (JO-JO^®^ France) and water provided *ad libitum*. Within this period, they were confirmed free of blood and gastrointestinal parasites through fecal floatation test and blood smear. The dogs were also vaccinated with anti-rabies, canine distemper, leptospirosis, hepatitis, parvovirus, and parainfluenza vaccines.

### Methodology

About 21 ml of venous blood was collected from each dog and 3 ml of each were dispensed into seven sterile plastic vacutainers and labeled thus for dog 1 (1A, 1B, IC, ID, 1E, 1F, and 1G), dog 2 (2A, 2B, 2C, 2D, 2E, 2F, and 2G), dog 3 (3A, 3B, 3C, 3D, 3E, 3F, and 3G), dog 4 (4A, 4B, 4C, 4D, 4E, 4F, and 4G), dog 5 (5A, 5B, 5C, 5D, 5E, 5F, and 5G), and dog 6 (6A, 6B, 6C, 6D, 6E, 6F, and 6G). The ratio of anticoagulant to blood was 1 ml of CPDA-1 to 7 ml of blood. The sample was collected within 20 min using standard aseptic procedures. The samples were thoroughly but gently mixed with the anticoagulant immediately after filling the tube. Blood in tubes suffixing A’s was analyzed within 30 min of blood collection, while blood in vacutainers suffixing B, C, D, E, F, and G’s was stored at 4°C in a solar refrigerator. Blood samples in plastic vacutainers suffixing B’s were assayed on day 1, C’s on day 2, D’s on day 3, E’s on day 7, F’s on day 10, and G’s on day 14. Parameters determined were packed cell volume (PCV), hemoglobin (Hgb) concentration, red blood cell (RBC) count, total white blood cell (TWBC) count, and platelet count (PC) [8]. Erythrocyte sedimentation rate (ESR) was also assayed [9]. Blood potency of hydrogen (pH) was determined (pHep tester, USA). Parameters assayed were determined in triplicate.

### Statistical analysis

The calculated values were entered into Excel sheet format for documentation and analysis. SPSS version 21 was used for statistical analysis. Mean, standard error of mean, and confidence intervals were determined for each hematological parameter. Data collected were statistically compared between groups using one-way analysis of variance. The least significant difference *post hoc* test was used to separate the variant means at p<0.05 as statistically significant.

## Results

On days 1, 2, and 7 post-storage (PS), mean PCV of CPDA-1 stored canine blood (SCB) was not significantly (p>0.05) different from PCV obtained from day 0 sample. However, PCV obtained from CPDA-1 SCB on days 3, 10, and 14 PS was significantly (p<0.05) lower than PCV of day 0 collection (Table-1). The mean Hgb concentration of CPDA-1 SCB on day 0 did not differ significantly (p>0.05) from that of days 1, 2, and 7 PS. However, mean Hgb concentration on days 10 and 14 was significantly lower (p<0.05) than those of days 0, 1, 2, and 7 PS (Table-1). There was no significant (p>0.05) difference in the mean RBC count of CPDA-1 SCB on days 1, 2, and 3 PS from that of day 0, while mean RBC count of CPDA-1 SCB on days 7, 10, and 14 PS was significantly (p<0.05) lower from that of days 0, 1, 2, and 3 (Table-1). During the first 2 days PS, the mean WBC count of CPDA-1 SCB did not differ significantly (p>0.05) from that of day 0. The WBC showed a downward trend on days 3 and 14 but an upward trend on days 7 and 10 days PS and differed significantly (p<0.05) from those of days 0, 1, and 2 (Table-2). During the 2-day period, the PC showed no significant (p>0.05) difference in the mean PC of CPDA-1 SCB from that of the baseline, day 0. However, from day 3 to day 14, there was a downward trend and the difference was statistically significant (p<0.05) from those of days 0, 1, and 2 (Table-2). On days 1, 2, and 3 PS, the ESR of CPDA-1 SCB was not significantly (p>0.05) different from ESR obtained from day 0 blood sample. However, ESR obtained from CPDA-1 SCB on days 7, 10, and 14 PS was significantly (p<0.05) lower than ESR of day 0 blood sample (Table-3). There was no significant (p>0.05) difference in the mean pH of CPDA-1 SCB on day 1 PS compared to that of day 0. However, the mean pH of CPDA-1 SCB on days 2, 3, 7, 10, and 14 PS was significantly (p<0.05) lower than the pH of day 0 (Table-3). The mean neutrophil count of CPDA-1 SCB on days 3 and 14 PS was significantly (p<0.05) lower than that of day 0. However, the mean neutrophil count of CPDA-1 SCB obtained on days 1, 2, 7, and 10 PS did not vary significantly (p>0.05) from that of day 0 (Table-4). The mean lymphocyte count of CPDA-1 SCB from day 0 did not differ significantly (p>0.05) from that of days 1, 2, 3, and 14 PS but was significantly (p<0.05) lower from that of days 7 and 10 PS (Table-4). There was no significant (p>0.05) difference in the mean basophil count (Table-4) of CPDA-1 SCB among the experimental days from that of day 0 PS. Furthermore, in comparing the mean monocyte and eosinophil count of the CPDA-1 SCB on days 1, 2, 3, 7, 10, and 14 PS, there was no significant (p>0.05) difference from that of day 0 (Table-4). On days 1, 2, 3, 7, 10, and 14 PS, the mean corpuscular Hgb concentration (MCHC) (Table-5) and MCH (Table-5) of CPDA-1 SCB did not vary significantly (p>0.05) from that of day 0. On days 1, 2, 3, 10, and 14 PS, the mean corpuscular volume (MCV) (Table-5) of the CPDA-1 SCB did not vary significantly (p>0.05) from that of day 0. However, the mean MCV of CPDA-1 SCB on day 7 PS was significantly (p<0.05) higher than that of day 0 as shown in Table-5.

**Table-1:** Changes in the mean PCV, Hgb, and RBC of CPDA-1 SCB PS of blood.

PS days	PCV (%)	Hgb (g/dL)	RBC (×10^6^/mL)
Day 0	37.89 (1.94)^a^	16.10 (1.49)^a^	5.06 (0.15)^a^
Day 1	37.50 (2.26)^a^	14.11 (4.76)^ab^	5.06 (0.09)^a^
Day 2	34.67 (3.08)^ab^	14.99 (1.27)^ab^	4.97 (0.05)^a^
Day 3	27.50 (2.26)^bc^	10.34 (0.96)^bc^	4.34 (0.54)^a^
Day 7	33.17 (3.19)^ab^	13.28 (4.26)^ab^	3.32 (0.80)^b^
Day 10	24.67 (8.08)^c^	11.00 (1.57)^bc^	2.83 (0.78)^b^
Day 14	11.50 (3.78)^d^	6.94 (1.81)^c^	1.82 (0.24)^d^

Different superscripts ^a,b,c,d^ in a column indicate a significant difference between the means at the level of probability: P<0.05. CPDA-1=Citrate phosphate dextrose adenine-1, PCV=Packed cell volume, RBC=Red blood cell, PS=Post-storage

**Table-2:** Changes in the mean TWBC and PC of CPDA-1 SCB PS of blood.

PS days	TWBC (×10^3^/mL)	PC (×10^3^/mL)
Day 0	14.12 (0.48)^b^	16.50 (4.04)^ab^
Day 1	14.13 (0.53)^b^	18.00 (2.19)^a^
Day 2	13.69 (0.32)^b^	15.67 (3.72)^abc^
Day 3	8.80 (3.42)^c^	1.83 (1.47)^c^
Day 7	18.83 (2.37)^a^	6.33 (2.80)^bc^
Day 10	18.38 (1.43)^a^	5.50 (7.01)^c^
Day 14	9.39 (1.85)^c^	2.33 (1.03)^d^

Different superscripts ^a,b,c,d^ in a column indicate a significant difference between the means at the level of probability: P<0.05. CPDA-1=Citrate phosphate dextrose adenine-1, TWBC=Total white blood cell, PC=Platelet count, PS=Post-storage

**Table-3:** Changes in the mean ESR and pH of CPDA-1 SCB at PS of blood.

Days	ESR	PH
Day 0	0.30 (0.10)^a^	5.87 (0.15)^a^
Day 1	0.25 (0.12)^ab^	5.75 (0.19)^ab^
Day 2	0.18 (0.08)^ab^	5.57 (0.10)^b^
Day 3	0.13 (0.06)^ab^	5.57 (0.11)^b^
Day 7	0.017 (0.03)^c^	4.88 (0.11)^c^
Day 10	0.00 (0.00)^c^	4.80 (0.21)^c^
Day 14	0.00 (0.00)^c^	4.02 (0.12)^d^

Different superscripts ^a,b,c,d^in a column indicate a significant difference between means at the level of probability: P<0.05. CPDA-1=Citrate phosphate dextrose adenine-1, ESR=Erythrocyte sedimentation rate, pH=Potency of hydrogen

**Table-4:** Changes in the mean DWBC of CPDA-1 SCB PS.

Days	N (×10^3^/µL)	L (×10^3^/µL)	B (×10^3^/µL)	M (×10^3^/µL)	E (×10^3^/µL)
Day 0	10.33 (1.90)^a^	3.54 (1.32)^b^	0.05 (0.12)^a^	0.17 (0.16)^a^	0.02 (0.06)^a^
Day 1	8.79 (1.39)^a^	4.78 (0.91)^ab^	0.05 (0.07)^a^	0.12 (0.10)^a^	016.(0.14)^a^
Day 2	8.55 (0.84)^a^	4.97 (1.03)^ab^	0.09 (0.11)^a^	0.12 (0.11)^a^	0.09 (0.14)^a^
Day 3	3.96 (1.67)^b^	4.62 (2.81)^ab^	0.02 (0.04)^a^	0.06 (0.06)^a^	0.08 (0.13)^a^
Day 7	11.08 (1.65)^a^	7.25 (2.08)^a^	0.00 (0.00)^a^	0.28 (0.21)^a^	0.21 (0.32)^a^
Day 10	11.04 (0.70)^a^	6.70 (2.12)^a^	0.06 (0.09)^a^	0.17 (0.24)^a^	0.13 (0.17)^a^
Day 14	3.76 (1.46)^b^	5.08 (0.88)^ab^	0.07 (0.08)^a^	0.07 (0.11)^a^	0.06 (0.09)^a^

Different superscripts ^a,b,c,d^in a column indicate a significant difference between means at the level of probability: P<0.05. CPDA-1=Citrate phosphate dextrose adenine-1

**Table-5:** Changes in the mean corpuscular values of CPDA-1 SCB PS.

Days	MCHC (g/dL)	MCH (Pg)	MCV (fL)
Day 0	42.51 (2.16)^a^	33.49 (3.29)^a^	72.39 (6.05)^a^
Day 1	42.07 (3.43)^a^	31.14 (2.83)^a^	74.03 (3.43)^a^
Day 2	43.54 (5.58)^a^	30.18 (2.43)^a^	69.80 (6.22)^a^
Day 3	37.99 (5.54)^a^	24.09 (3.68)^a^	63.81 (6.28)^a^
Day 7	40.10 (12.76)^a^	45.77 (32.50)^a^	106.47 (34.77)^b^
Day 10	53.35 (32.19)^a^	42.65 (17.75)^a^	85.78 (15.51)^ab^
Day 14	66.23 (27.81)^a^	39.17 (12.72)^a^	63.47 (18.10)^a^

Different superscripts ^a,b,c,d^ in a column indicate a significant difference between means at the level of probability: P<0.05. CPDA-1=Citrate phosphate dextrose adenine-1, MCHC=Mean corpuscular Hgb concentration, MCV=Mean corpuscular volume, Hgb=Hemoglobin

## Discussion

The stability of Nigerian indigenous dog blood stored with CPDA-1 anticoagulated vacutainers was determined in this study. Specifically, variations in hematological parameters, blood pH, and ESR were used to assess the stability of the stored blood.

CPDA-1 provides substrate from which RBCs can synthesize ATP during storage and also improves viability. The dextrose supports continuing ATP generation by glycolytic pathway, while the citrate prevents coagulation by binding ionized calcium which is required for coagulation cascade. In this study, the blood samples were stored in plastic vacutainers containing CPDA-1 and were preserved in a refrigerator (at 4°C) for 14 days. The results obtained showed that the mean values of PCV, Hgb concentration, and RBC count were not significantly different from that of the baseline values up to day 2 PS. The outcomes of some researchers on the stability of blood at 4°C in different animals with variants of anticoagulants have been properly documented. However, there is no information existing on the effects of CPDA-1 on the viability of stored Nigerian indigenous dog’s blood. The reports of other studies with ethylenediaminetetraacetic acid (EDTA) stored blood in ovine [10], humans [11], turkey [12], and fish [13] were in agreement with our findings. Lower temperature storage assisted in prolonging the life span of the studied analytes [12].

This study also showed a significant drop in PCV and Hgb concentration on day 3, while both analytes increased by day 7 PS. However, EDTA-induced erythrocyte swelling by 72-h PS has been reported in fish [14] and with human’s blood [11]. Erythrocyte swelling may occur due to an increase in pCO_2_ and acidification after treatment with acidic EDTA salt. It has been suggested that this swelling may lead to erythrocyte hemolysis and membrane distortion affecting RBC and other hematological parameter readings [13,14].

This study showed that MCV values were unchanged up to day 3 PS, while the value obtained by day 7 of storage was significantly higher than recorded values on days 0, 1, 2, 3, and 14. Previously, it had recorded stable MCV of human blood preserved at 2-6°C with EDTA anticoagulant for 72 h [9,15-17]. Furthermore, increased intracellular sodium content affects cell volume and shape such that the MCV of stored red cells is increased after days to weeks of storage [18]. This could be the attributed reason for the increase in the MCV on days 7 and 10 PS. A decrease in MCH and MCHC is caused by hematocrit increase when the Hgb level is still stable [3]. The above reason could be the reason for the obtained result in the MCV value. Furthermore, as commonly known, MCHC is a parameter calculated by the ratio of Hgb to hematocrit multiplied by 100. Changes in the components of this equation will also lead to a change in MCHC value. The above factor could be responsible for unchanged MCH and MCHC as obtained during the course of the work.

The result of TWBC and PC showed that there was decreased leukocyte count and PC by day 3 PS. Studies done with human blood stored with CPDA-1 also showed the same trend with our finding [19]. Furthermore, some researchers reported instability of TWBC and PC of human blood 24 h PS with EDTA and CPDA-1 [20]. They suggested that the changes observed were most likely due to the degenerative changes known to occur as cells age.

From this study, the blood acid-base balance (blood pH) of Nigerian local dogs stored in CPDA-1 was stable for 24-h PS at 4°C. In previous studies, it has been recorded that the blood acid-base parameters were stable in the blood samples between 15 min and 2 h in man [21], up to 24 h in cattle [22], and up to 6 h after 4°C refrigeration in goat [23]. The pH values of ovine blood samples kept in freeze-dried lithium heparin at 4°C were also found to be suitable for clinical purposes up to 48 h [24]. It could be suggested that drop in blood pH could be due to the continuity metabolism in the form of oxygen consumption as a result of anaerobic metabolism with the generation of carbon dioxide in the tricarboxylic acid cycles [25] or accumulation of lactic acid due to glycolysis [22]. Hence, it is assumed that the drop in blood pH can be attributed to the anaerobic metabolism in the blood by WBCs causing consumption of oxygen in blood samples stored under *in vitro* anaerobic condition [22].

Furthermore, the type of syringe used for sampling has been incriminated to influence alterations in the levels of oxygen levels [21]. It has been revealed that oxygen level increases by the diffusion of oxygen through the plastic wall while carbon dioxide does not diffuse [4]. A decrease in oxygen values has been found in blood samples with high leukocyte counts [26] and in samples with anemia [27]. Since the initial mean WBC and RBC count, as well as Hgb concentrations of the blood samples, were within normal range between 24 and 72 h in the present study, a decrease in oxygen values may have been due to the release of oxygen from Hgb as a result of the reduction in blood pH [22] and the diffusion of oxygen through the plastic vacutainer [21,29].

ESR remained unchanged for 72-h PS as observed from the finding. Its increase is usually associated with conditions of cellular degeneration, tissue damage, and inflammation. ESR test is, however, of great importance in monitoring the course of a disease, particularly in evaluating the response of the individual to cellular degeneration as well as inflammatory or necrotic processes [28]. The speed is increased in blood samples from animals suffering from inflammatory diseases, in which there are tissue necrosis and degeneration. In such animals, this increased speed of sinking (sedimentation rate) had been attributed to increases in plasma fibrinogen and consequent changes that occur on the erythrocyte surfaces that cause red cells to form relatively larger and more aggregates (rouleaux) which sink or fall more rapidly [9]. From the study, ESR and RBC count was stable for about 72-h PS. We assumed that the ESR did not increase for this period of time because the blood was harvested from healthy dogs.

## Conclusion

In summary, this study demonstrated that SCB of Nigerian indigenous dogs with CPDA-1 concentration of 1 ml:7 ml (required for human blood preservation) was stable for 48-72 h at the limits of approved cold storage time, 4°C. It could, therefore, be suggested that rapid degeneration of SCB was as a result of the plastic vacutainer used. Hence, there is a need to design small plastic vacutainers for collection and storage of blood for transfusion purposes in small breeds of dogs like our Nigerian indigenous dog breeds.

## Authors’ Contributions

URI and OHN conceived the idea. OHN wrote the first draft of the manuscript. OHN, URI, and NTO were involved in the design and carried out the experimental work. The manuscript was critically read, revised, and approved for submission by all the authors.
